# *Giardia* Cyst Wall Protein 1 Is a Lectin That Binds to Curled Fibrils of the GalNAc Homopolymer

**DOI:** 10.1371/journal.ppat.1001059

**Published:** 2010-08-19

**Authors:** Aparajita Chatterjee, Andrea Carpentieri, Daniel M. Ratner, Esther Bullitt, Catherine E. Costello, Phillips W. Robbins, John Samuelson

**Affiliations:** 1 Department of Molecular and Cell Biology, Boston University Goldman School of Dental Medicine, Boston, Massachusetts, United States of America; 2 Department of Biophysics and Physiology, Boston University School of Medicine, Boston, Massachusetts, United States of America; 3 Department of Biochemistry, Boston University School of Medicine, Boston, Massachusetts, United States of America; University of California Los Angeles, United States of America

## Abstract

The infectious and diagnostic stage of *Giardia lamblia* (also known as *G. intestinalis* or *G. duodenalis*) is the cyst. The *Giardia* cyst wall contains fibrils of a unique β-1,3-linked N-acetylgalactosamine (GalNAc) homopolymer and at least three cyst wall proteins (CWPs) composed of Leu-rich repeats (CWP_LRR_) and a C-terminal conserved Cys-rich region (CWP_CRR_). Our goals were to dissect the structure of the cyst wall and determine how it is disrupted during excystation. The intact *Giardia* cyst wall is thin (∼400 nm), easily fractured by sonication, and impermeable to small molecules. Curled fibrils of the GalNAc homopolymer are restricted to a narrow plane and are coated with linear arrays of oval-shaped protein complex. In contrast, cyst walls of *Giardia* treated with hot alkali to deproteinate fibrils of the GalNAc homopolymer are thick (∼1.2 µm), resistant to sonication, and permeable. The deproteinated GalNAc homopolymer, which forms a loose lattice of curled fibrils, is bound by native CWP1 and CWP2, as well as by maltose-binding protein (MBP)-fusions containing the full-length CWP1 or CWP1_LRR_. In contrast, neither MBP alone nor MBP fused to CWP1_CRR_ bind to the GalNAc homopolymer. Recombinant CWP1 binds to the GalNAc homopolymer within secretory vesicles of *Giardia* encysting *in vitro*. Fibrils of the GalNAc homopolymer are exposed during excystation or by treatment of heat-killed cysts with chymotrypsin, while deproteinated fibrils of the GalNAc homopolymer are degraded by extracts of *Giardia* cysts but not trophozoites. These results show the Leu-rich repeat domain of CWP1 is a lectin that binds to curled fibrils of the GalNAc homopolymer. During excystation, host and *Giardia* proteases appear to degrade bound CWPs, exposing fibrils of the GalNAc homopolymer that are digested by a stage-specific glycohydrolase.

## Author Summary

While the walls of plants and fungi contain numerous sugar homopolymers (cellulose, chitin, and β-1,3-glucans) and dozens of proteins, the cyst wall of *Giardia* is relatively simple. The *Giardia* wall contains a unique homopolymer of β-1,3-linked N-acetylgalactosamine (GalNAc) and at least three cyst wall proteins (CWPs), each of which is composed of Leu-rich repeats and a C-terminal Cys-rich region. The three major discoveries here are: 1) Fibrils of the GalNAc homopolymer are curled and form a lattice that is compressed into a narrow plane by bound protein in intact cyst walls. 2) Leu-rich repeats of CWP1 form a novel lectin domain that is specific for fibrils of the GalNAc homopolymer, which can be isolated by methods used to deproteinate fungal walls. 3) A cyst-specific glycohydrolase is able to degrade deproteinated fibrils of the GalNAc homopolymer. We incorporate these findings into a new curled fiber and lectin model of the intact *Giardia* cyst wall and a protease and glycohydrolase model of excystation.

## Introduction

*Giardia lamblia*, which is a deeply divergent protist, causes diarrhea in the developing world where hygiene is inadequate to block its transmission by the fecal-oral route [1]–[3]. In addition, some two million Americans are infected each year with *Giardia*, which is present in streams and lakes or is transmitted in day-care centers [4]. *Giardia* is then an important pathogen in both developing and developed countries.

The infectious and diagnostic stage of *Giardia* is the quadranucleate cyst [5]. Three abundant cyst wall proteins (CWP1, CWP2, and CWP3) have *N*-terminal Leu-rich repeats (LRRs) and a C-terminal Cys-rich region (CRR) [6]–[9]. Cys-rich CWP1 is the target for diagnostic monoclonal antibodies to *Giardia* in clinical specimens, and anti-CWP1 antibodies reduce excystation of *Giardia in vitro* [10], [11]. CWP2, which has an additional positively charged domain at its C-terminus, has been used to immunize mice and reduce cyst formation by *Giardia* [12]. The three *Giardia* CWPs have relatively few sites for *N*-linked glycosylation, so that wheat germ agglutinin (WGA), which binds to the very short *N*-glycan of *Giardia* (GlcNAc_2_), predominantly stains membranes closely apposed to the cyst wall rather than the wall itself [13]–[15]. A fourth cyst wall protein (HCNCp) is part of a new family of Cys-rich, non-VSP proteins of *Giardia* [16]. In addition, a family of proteins referred to as EGF-like cyst proteins (EGFCPs), which contain a series of Cys-rich repeats, are targeted to *Giardia* cyst walls [17].

CWPs are present in encystation-specific secretory vesicles (ESVs), which are part of a Golgi-like compartment that lacks membrane stacks and luminal glycosyltransferases but is sensitive to Brefeldin A [18]. The positively charged domain at the C-terminus of CWP2 is important for biogenesis of ESVs [19]. Selective condensation drives portioning and sequential secretion of cyst wall proteins, so that CWP1 and the major portion of CWP2 are added first to the cyst wall followed by CWP3 [20].

*Giardia* cysteine proteinases are necessary for encystation and excystation, while host proteases (trypsin and/or chymotrypsin) are required for excystation [21]–[23]. ESVs and cyst wall formation are interrupted by dithiothreitol (DTT) that blocks disulfide formation within Cys-rich C-terminal domains of CWPs and blocks polymerization of CWPs [24]. Cyst wall formation is also dependent upon isopeptide bonds formed in CWPs by a novel transglutaminase activity [25]. Finally, protein phosphatases are involved in cyst wall formation [26], [27].

Pioneering studies of Edward Jarroll and colleagues have shown that the sugar homopolymer in *Giardia* cyst walls is composed of β-1,3-linked N-acetylgalactosamine (GalNAc) rather than chitin (β-1,4-linked GlcNAc), as previously suggested [28]. Electron microscopic studies demonstrate the deposition of fibrils of the GalNAc homopolymer onto the surface of encysting *Giardia*, as well as within intracellular vesicles [29], [30]. However, the GalNAc homopolymer has not been obtained free of protein contamination, so that its structure in the absence of protein has not been visualized. In addition, no reagents (lectins or antibodies) have been identified for labeling the GalNAc homopolymer, which does not stain with GalNAc-binding plant lectins [13]. The GalNAc homopolymer is made from cytosolic UDP-GalNAc by a synthase [31].

The walls of fungi, plants, and *Dictyostelium* contain multiple layers, more than one sugar polymer, and many proteins, and so these walls likely do not represent a good model for the relatively simple cyst wall of *Giardia* [32]–[34]. In contrast, the cyst wall of *Entamoeba histolytica*, the protist that causes amebic dysentery and liver abscess, is relatively simple [35], [36]. The amebic wall is composed of chitin and three unique chitin-binding lectins, which degrade chitin (chitinase), cross-link fibrils (multivalent Jacob lectins), and self-aggregate to make the amebic wall impermeable to small molecules (Jessie lectins) [37]–[39].

In an effort to better understand how the *Giardia* cyst wall is assembled during encystation, we asked the following questions:

- Can we use methods used to isolate chitin and glucans from fungal walls (strong alkali and high temperatures) to deproteinate cyst walls of *Giardia* and isolate fibrils of the GalNAc homopolymer [40]? If so, what do the fibrils look like?
- Do native CWPs, which are released with non-ionic detergent from encysting *Giardia*, bind to deproteinated fibrils of the GalNAc homopolymer? If so, can we use recombinant maltose-binding fusion-proteins (MBP) to determine whether the lectin domain of CWP1 is present in the *N*-terminal Leu-rich repeats (CWP1_LRR_) or in the *C*-terminal Cys-rich region (CWP1_CRR_) [39], [41]?
- Can we use recombinant CWP1 to determine whether the GalNAc homopolymer is made at the plasma membrane, as described for fungal chitin, or within intracellular vesicles as described for chitin of *Entamoeba* [39], [42]?
- Can we provide evidence that an endogenous *Giardia* glycohydrolase (comparable to chitinases of *Entamoebae*) degrades fibrils of the GalNAc homopolymer during excystation (37)?

## Results

### Intact *Giardia* cyst walls are thin, brittle, and impermeable to small molecules

**Figure 1:**
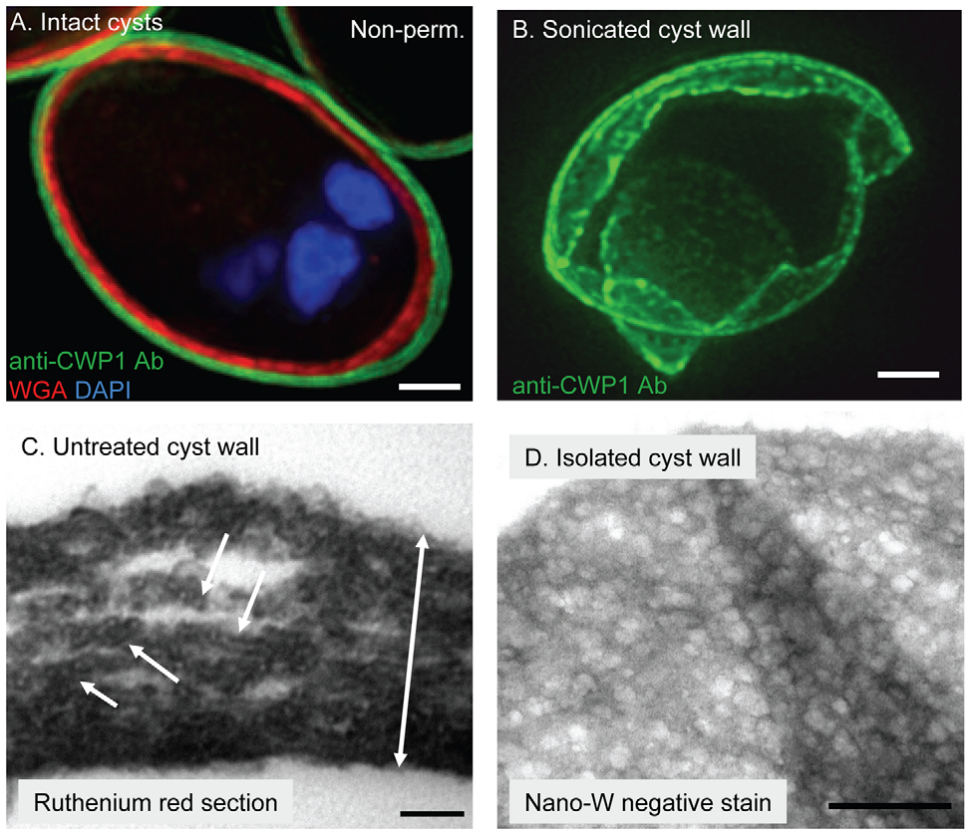
In intact *Giardia* cyst walls, curled fibrils of the GalNAc homopolymer form a protein-coated lattice that is restricted to a narrow plane. A. Cross-section by deconvolving microscopy shows intact cysts label well with anti-CWP1 monoclonal antibody (green). Unless cysts were frozen and thawed, WGA (red), which intensely labels *N*-glycans present in membrane glycoproteins closely apposed to the cyst wall, does not penetrate (for example, cyst labeled non-perm. in upper right corner of A). Nuclei are stained blue with DAPI. B. Three-dimensional reconstruction by deconvolving microscopy shows that a cyst wall broken by sonication and then labeled with anti-CWP1 antibodies (green) is thin and shows sharp lines of fracture. C. TEM of a thin section of ruthenium red-stained *Giardia* cysts shows the intact cyst wall is thin (two-headed arrow is 0.4 microns long). Fibers of the GalNAc homopolymer are coated with a linear array of oval-shaped protein complexes (arrows). D. TEMs of a *Giardia* cyst wall isolated on a sucrose gradient and then negatively stained with NANO-W shows a two-dimensional honey-comb appearance, in which curled fibrils of the GalNAc homopolymer are held in a narrow plane by adherent CWPs. Note folds in the sheet-like cyst wall. Bars (A and B) are 1 micron. Bar (C) is 100 nm. Bar (D) is 500 nm.

*Giardia* cyst walls were visualized well with an anti-CWP1 monoclonal antibody (Fig. 1A) [7], [10]. *Giardia* cyst walls are impermeable, so we froze and thawed cysts multiple times in order to label nuclei with DAPI and label *N*-linked glycans that are present for the most part in membranes closely apposed to the cyst wall with WGA (Fig. 1A) [15]. *Giardia* cyst walls are brittle and so fracture into multiple fragments or shards with sharp edges when sonicated (Fig. 1B). *Giardia* cyst walls, which stain well with the poly-cationic dye ruthenium red, are 0.3 to 0.6 µm thick (depending upon the compactness of the fibrils) when viewed on cross-section with the transmission electron microscope (TEM) (Fig. 1C) [43]. Fibrils of the GalNAc homopolymer are covered with linear arrays of oval-shaped protein complexes that have a uniform appearance (Fig. 1C). These oval-shaped protein complexes, which are too big to represent a single CWP, are lost when cyst walls are deproteinated with NaOH (see next section).

Fibrils of the GalNAc homopolymer were also visualized when *Giardia* cyst walls were isolated on sucrose gradients, negative-stained with NANO-W (an organo-tungstate compound that is weakly penetrating and so highlights superficial structures), and viewed with TEM (Fig. 1D) [44]. In these cyst wall preparations, curled fibrils of the GalNAc homopolymer form a lattice that is pressed into thin sheets and has the appearance of a two-dimensional honey comb. Curled fibrils of the GalNAc homopolymer are also present in cyst walls deproteinated by NaOH-treatment, but the curled fibrils no longer form a thin sheet (see next section).

### Treatment of *Giardia* cyst walls with hot alkali removes cyst wall proteins (CWPs)

**Figure 2:**
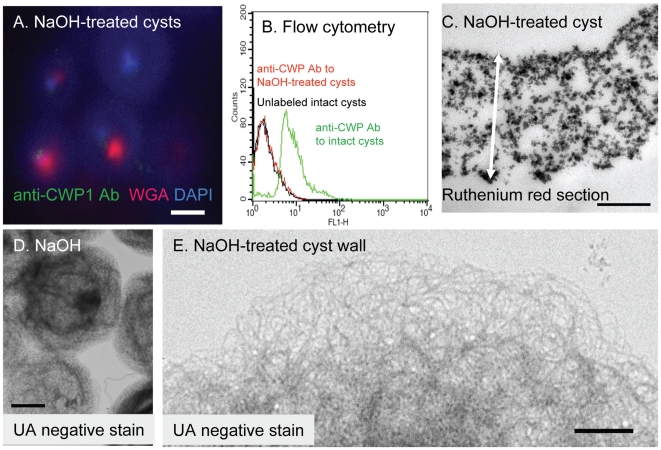
The deproteinated GalNAc homopolymer (NaOH-treated cyst wall) is a loose, thick-walled lattice of curled fibrils that maintains a hollow, spherical shape. A. Deconvolving microscopy shows NaOH-treatment removes cyst wall proteins, so that there is no longer any binding of anti-CWP1 antibodies (green). WGA (red) labels some denatured parasite glycoproteins that are not part of the cyst walls, while DAPI (blue) stains residual nuclei acid. B. Flow cytometry shows that binding of anti-CWP1 antibodies to intact cyst walls (green) is completely removed by NaOH treatment (red). A control with unlabeled intact cysts is shown in black. C. A TEM of a thin section of ruthenium red-stained cyst wall after treatment with NaOH shows fibrils of the GalNAc homopolymer are no longer coated with oval-shaped protein complexes, while the cyst wall is markedly thickened (two-headed arrow is 1.2 microns long). D and E. TEM and negative staining with uranyl acetate (UA) shows NaOH-treated cyst walls of *Giardia* are composed of a loose lattice of 5 nm thick, curled fibrils that generally maintain the hollow spherical shape of the cyst wall. NaOH-treated cyst walls do not stain with NANO-W (data not shown). Bar (A) is 1 micron. Bars (C and E) are 100 nm. Bar (D) is 5 microns.

**Figure 3:**
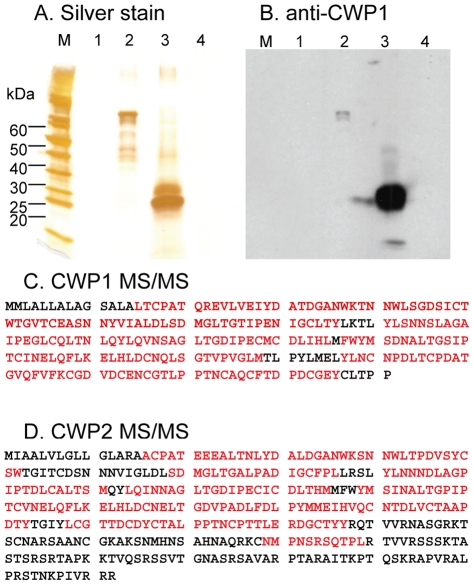
Binding of CWP1 and CWP2 to deproteinated fibrils of the GalNAc homopolymer. Silver stain (A) and Western blot with anti-CWP1 antibody (B) show that cyst walls treated with NaOH (lane 1 in each figure) contain no detectable protein (see Fig. 2 for the appearance of deproteinated fibrils of the GalNAc homopolymer). In contrast, recombinant MBP-CWP1_FL_ made in bacteria (lane 2 in A and B) binds well to deproteinated fibrils of the GalNAc homopolymer. Native CWP1, which is present in extracts of encysting *Giardia* (lane 3 in A and B), also binds to deproteinated fibrils of the GalNAc homopolymer. In contrast, no trophozoite proteins bind to deproteinated cyst walls (lane 4 in A and B). C. Mass spectrometry shows that CWP1 present in extracts of encysting *Giardia* but minus the N-terminal signal peptide binds to deproteinated fibrils of the GalNAc homopolymer (peptide coverage is 86%). D. Mass spectrometry shows that CWP2 present in extracts of encysting *Giardia* but minus the N-terminal signal peptide and the C-terminal positively charged tail also binds to deproteinated fibrils of the GalNAc homopolymer (peptide coverage is 56%). These results show that NaOH removes proteins from *Giardia* cyst walls and show that the fibrils of the GalNAc homopolymer bind native and recombinant CWP1, as well as native CWP2.

Because preparations of the GalNAc homopolymer from cyst walls of *Giardia* that have been treated with proteases and detergents still contain substantial quantities of contaminating protein [28], we chose hot alkali treatment that is frequently used to isolate chitin and β-1,3-glucans from deproteinated fungal walls [40]. We isolated fibrils of the GalNAc homopolymer, which generally maintains the hollow spherical shape of cyst walls, by boiling whole *Giardia* cysts in 0.75 to 1 N NaOH for 1 to 2 hrs (Fig. 2). The purity of the deproteinated fibrils of the GalNAc homopolymer (synonym for NaOH-treated cyst walls) was demonstrated in three ways:

- While both intact cysts and shards of the cyst wall label strongly with an anti-CWP1 monoclonal antibody (Figs. 1A and 1B), NaOH-treated cyst walls do not label with the anti-CWP1 antibody (Fig. 2A). The results with deconvolving microscopy were confirmed by flow cytometry (Fig. 2B). The opposite results occur when intact and NaOH-treated cyst walls are labeled with recombinant CWP1, which binds to fibrils of the GalNAc homopolymer (see below).
- Only GalN and GalNAc are released by acid hydrolysis of NaOH-treated *Giardia* cyst walls. These sugars were shown by gas chromatography and mass spectrometry (GC-MS) and by high performance anion exchange chromatography (HPAEC) (Fig. S1 in the Supplemental materials). These results are in agreement with release of monomers, dimers, and short oligosaccharides of β-1,3-linked GalNAc by partial acid hydrolysis of *Giardia* cyst walls that were deproteinated with SDS and proteases [21].
- NaOH-treated cyst walls do not contain protein, as judged by a BCA assay and by SDS-PAGE followed by silver-staining or Western blotting with anti-CWP1 antibody (lane 1 in Figs. 3A and 3B). Positive controls for the BCA-assays, silver stains, and Western blots were made by examining NaOH-treated cyst walls that had been incubated with native *Giardia* CWPs or with MBP fusion-proteins containing *Giardia* CWP1 (lanes 2 and 3, respectively, in Figs. 3A and 3B and see below).
- We cannot rule out, however, the possibility that treatment with strong base may cleave certain covalent linkages between chains of GalNAc homopolymers and/or remove other attached sugars.

### The deproteinated GalNAc homopolymer is a loose, thick-walled lattice of curled fibrils

**Figure 4:**
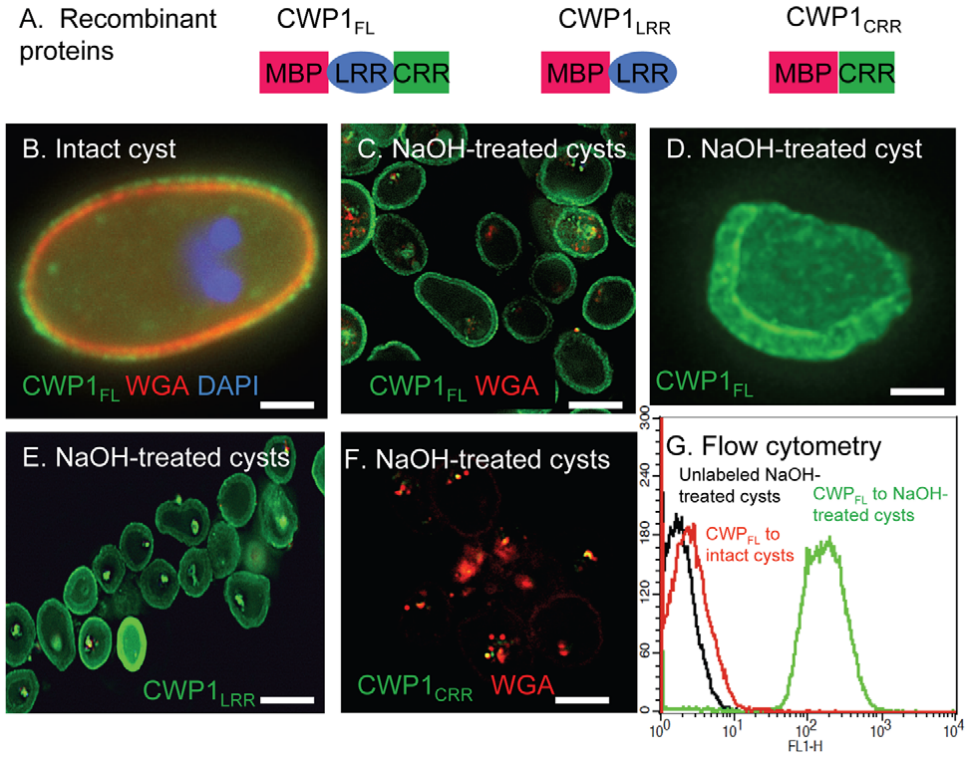
*N*-terminal Leu-rich repeats of *Giardia* CWP1 (CWP1_LRR_) form a lectin domain that binds to deproteinated fibrils of the GalNAc homopolymer. A. Each recombinant protein used to identify the lectin domain of CWP1 has MBP at the *N*-terminus. At the *C*-terminus, CWP1_LRR_ contains the Leu-rich repeats of CWP1; CWP1_CRR_ contains the Cys-rich region; while full-length CWP1 (CWP1_FL_) contains both domains. B. A representative deconvolving micrograph shows CWP1_FL_ (green) binds in a punctate pattern to the wall of an intact cyst, while WGA (red) and DAPI (blue) appear as in Fig. 1A. C. CWP1_FL_ (green) strongly labels NaOH-treated cyst walls (deproteinated GalNAc homopolymer), while there is minimal labeling of residual glycoproteins with WGA. D. A shard of a *Giardia* cyst wall made by sonication, treatment with NaOH, and then incubation with CWP1_FL_ (green) is >1 µm thick. E. CWP1_LRR_ (green) also labels deproteinated fibrils of the GalNAc homopolymer. F. In contrast, there is no binding of CWP1_CRR_ (green) to NaOH-treated cyst walls. Again the only staining is with WGA that binds to glycoproteins that are not part of the cyst wall. There is no binding of unfused MBP to *Giardia* deproteinated fibrils of the GalNAc homopolymer (data not shown). G. Flow cytometry shows that binding of CWP1_FL_ to intact cyst walls (black) is much less than the binding of the same probe to NaOH treated walls (green). A control with unlabeled NaOH-treated cysts is shown in red. All micrographs are cross-sections with the exception of C, which is a three-dimensional reconstruction. Bars (B and D) are 2 microns. Bar (C, E, and F) are 10 microns.

NaOH-treated *Giardia* cysts stick to one another, most likely by interlocking fibrils of the GalNAc homopolymer that were exposed by removal of cyst wall proteins (Figs. 2D and 2E). The deproteinated fibrils of the GalNAc homopolymer, which are five nm thick and curled, are no longer coated by oval-shaped protein complexes. The fibrils of the deproteinated GalNAc polymer form a loose lattice in the general shape of untreated cyst walls, but they are no longer pressed into a narrow two-dimensional sheet. Instead NaOH-treated cyst walls are substantially thicker than untreated cyst walls (compare Fig. 1C to Fig. 2C and compare Fig. 2B to Fig. 4D). In addition, NaOH-treated cysts are permeable to WGA and DAPI that bind to remnant coagulated protein and DNA, respectively, in poorly washed preparations (Fig. 2A). NaOH-treated cysts are no longer brittle and so cannot be broken by sonication (data not shown). The fragment of the cyst wall shown in Fig. 4D was prepared by sonicating intact cysts that were then treated with hot alkali prior to labeling with recombinant CWP1 (see description in next section).

Together these results suggest that *Giardia* cyst wall proteins, which bind in linear arrays of oval-shaped protein complexes to fibrils of the GalNAc homopolymer, contribute to the thinness, brittleness, and impermeability of the cyst wall. However, deproteinated curled fibrils of the GalNAc homopolymer are capable of maintaining the hollow spherical shape of the cyst wall.

### Native CWP1 and CWP2 from encysting *Giardia* bind to deproteinated fibrils of the GalNAc homopolymer

We used the deproteinated fibrils of the GalNAc homopolymer to pull down proteins of encysting *Giardia* that have lectin (carbohydrate-binding) activity. Native CWP1, which was demonstrated by silver staining of SDS-PAGE, Western blotting with anti-CWP1 antibodies, and mass spectrometry of peptides released by chymotrypsin, bound strongly to fibrils of the GalNAc homopolymer (Figs. 3A to 3C). In particular, there was 96% peptide coverage of CWP1 (less the signal peptide) in mass spectrograms of encystation-specific proteins of *Giardia* binding to deproteinated fibrils of the GalNAc homopolymer. As a negative control, no trophozoite proteins bound to the deproteinated fibrils of the GalNAc homopolymer (lane 4 in Figs. 3A and 3B).

Native CWP2 (less the signal peptide), also demonstrated by mass spectrometry, bound strongly to fibrils of the GalNAc homopolymer (Fig. 3D). There was much less peptide coverage of the C-terminus of CWP2, consistent with previous observations that ∼60 amino acids are cleaved from the C-terminus by an endogenous protease [20], [21]. The peptide coverage of CWP3 bound to deproteinated fibrils of the GalNAc homopolymer (12%) was much less than those for CWP1 (86%) and CWP2 (56%). This result suggests CWP3 is much less abundant in lysates of encysting *Giardia* and/or binds much less well to fibrils of the GalNAc homopolymer. Other proteins identified by mass spectrometry showed many fewer peptides, included cytoskeletal proteins, fermentation enzymes, and chaperones. All of these proteins are cytosolic in origin and are likely contaminants.

### The Leu-rich repeats of *Giardia* cyst wall protein 1 (CWP1_LRR_) form a lectin domain that binds fibrils of the GalNAc homopolymer

**Figure 5:**
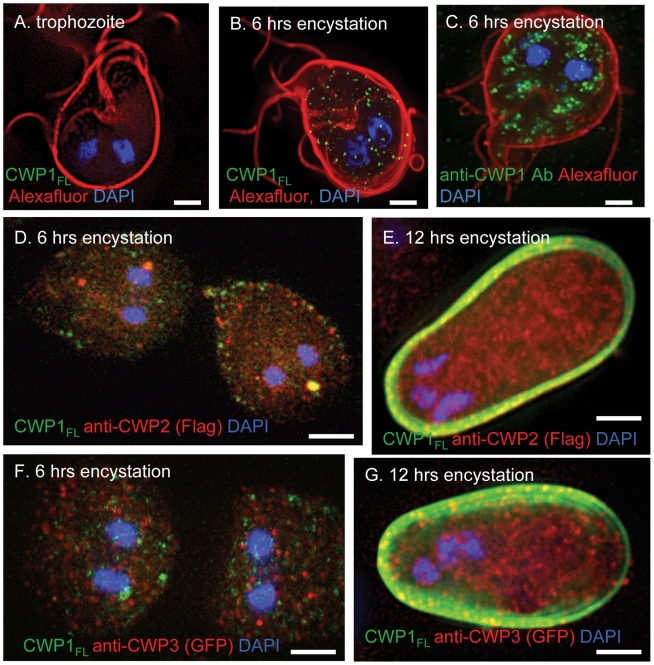
The GalNAc homopolymer is made within small vesicles of encysting *Giardia* and is added early to the cyst wall. A. A representative deconvolving micrograph shows there is no synthesis in *Giardia* trophozoites of the GalNAc homopolymer that is detected with an MBP-CWP1 full-length fusion-protein (CWP1_FL_) (green). The plasma membrane is labeled red with Alexafluor. B. *Giardia* encysting for 6 hrs shows an extensive sets of vesicles labeled with CWP1_FL_ (green), which binds to the GalNAc homopolymer. C. These vesicles are smaller and less clumped than those labeled green with anti-CWP1 antibodies in a parallel set of *Giardia* encysting for 6 hrs. D. Double-labeling experiment of a transformed *Giardia* expressing epitope-tagged CWP2 shows the GalNAc homopolymer (labeled green with recombinant CWP1_FL_) is present in vesicles of parasites encysting for 6 hrs that are for the most part distinct from ESVs containing CWP2 (labeled red with an antibody to the FLAG-tag). E. After 12 hrs encystation, the GalNAc homopolymer is present in the cyst wall, while the bulk of CWP2 remains in ESVs. F. Double-labeling experiment of a transformed *Giardia* expressing epitope-tagged CWP3 shows the GalNAc homopolymer is present in vesicles of parasites encysting for 6 hrs that are for the most part distinct from ESVs containing CWP3 (labeled red with an antibody to GFP). G. After 12 hrs, the GalNAc homopolymer is present in the cyst wall, while the bulk of CWP3 remains in ESVs. All figures are three-dimensional micrographs. Bars (A to G) are 2 microns.

To determine which domain of CWP1 contains the lectin activity that binds to fibrils of the GalNAc homopolymer, we made MBP fusion-proteins containing at the *C*-terminus either the full-length CWP1 less the signal peptide (CWP1_FL_), the *N*-terminal Leu-rich repeats of CWP1 less the signal peptide (CWP1_LRR_), or the *C*-terminal Cys-rich regions of CWP1 (CWP1_CRR_) (Fig. 4A) [41]. CWP1_FL_, which weakly labels intact cyst walls of *Giardia* (Fig. 5B), binds strongly to deproteinated fibrils of the GalNAc homopolymer (Figs. 4C and 4D). The CWP1_FL_-labeled fibrils of the GalNAc homopolymer are thick-walled, and the edges of the walls are mostly densely labeled, producing a “two-layered appearance.” This two-layered appearance was not reproduced in transmission micrographs of sections of deproteinated walls (Fig. 2C) or negative stains of the deproteinated walls (Figs. 2D and 2E), suggesting the two layers may explained by an increased density of curled fibrils at the outer and inner surfaces of the deproteinated walls. These deconvolving microscopy results were confirmed by flow cytometry (Fig. 4G) and by SDS-PAGE and Western blotting (lane 2 in Figs. 3A and 3B). While CWP1_LRR_ labels NaOH-treated cyst walls as strongly as does CWP1_FL_ (Fig. 4E), CWP1_CRR_ does not bind at all to deproteinated fibrils of the GalNAc homopolymer (Fig. 4F).

These results show that the CWP1_LRR_ contains a lectin domain that binds to deproteinated fibrils of the GalNAc homopolymer and suggest the use of recombinant CWP1_FL_ to visualize the GalNAc homopolymer during encystation (see next section). Recombinant CWP1_FL_ is an important new tool for localizing fibrils of the GalNAc homopolymer, as GalNAc-binding plant lectins (e.g. *Maclura pomifera* agglutinin, also known as MPA) fail to bind to the deproteinated fibrils of the GalNAc homopolymer (data not shown) [13]. Conversely, recombinant CWP1_FL_ does not bind to oocyst walls of *Cryptosporidia* that label strongly with MPA (data not shown) [45].

Recombinant MBP-CWP2 and MBP-CWP3 each failed to bind to the GalNAc homopolymer (data not shown). Because we cannot rule out the possibilities that MBP-CWP1_CRR_, MBP-CWP2, and MBP-CWP3 fusion-proteins are not well-folded, it is possible that these experiments failed for technical reasons and do not reflect the situation *in vivo* (see pull down of native CWP2 by the GalNAc homopolymer in Fig. 3D). Because native CWP1 is also present in extract of encysting *Giardia*, it is possible that native CWP2 is binding to CWP1 rather than to the GalNAc homopolymer. In particular, intermolecular disulfide bonds have been shown between CWP1 and CWP2 [24].

### The GalNAc homopolymer is synthesized within small vesicles in encysting *Giardia* and is an early component of the cyst wall

These experiments used recombinant CWP1_FL_ to identify the GalNAc homopolymer during encystation of *Giardia in vitro*. *Giardia* trophozoites, the surfaces of which were labeled with an Alexafluor dye, do not stain with CWP1_FL_ (Fig. 5A) [46]. The GalNAc homopolymer, which was detected with recombinant CWP1_FL_, is present in small vesicles throughout the cytosol of *Giardia* encysting for 6 hrs (Fig. 5B). CWP1, which was detected by a monoclonal antibody (Fig. 5C), is present in ESVs [5], [6], [19], [20]. ESVs tend to be larger and located closer to the nucleus than vesicles that contain the GalNAc homopolymer. These results confirm TEM visualization of fibrils of the GalNAc homopolymer within secretory vesicles of encysting *Giardia* that are then deposited on the surface of encysting organisms [29], [30].

Because we used recombinant CWP1_ FL_ to visualize the GalNAc homopolymer, we could not use a double-label with the anti-CWP1 antibody. However, we performed double-labels with recombinant CWP1_ FL_ and antibodies that bound to epitope-tagged CWP2 and CWP3 in transformed *Giardia* that were encysting *in vitro* (Figs. 5D to 5G) [20]. For the most part, vesicles labeled with recombinant CWP_FL_ do not also label with antibodies that bind to CWP1 and CWP2. Further recombinant CWP1 labels cyst walls when antibodies to CWP2 and CWP3 predominantly labeled ESVs in the interior of *Giardia*. These results suggest that the GalNAc homopolymer is made in vesicles distinct from ESVs and is secreted early onto the surface of encysting *Giardia*, as has also been shown by the scanning electron microscope [29].

### During excystation *in vitro*, CWPs appear to be digested by proteases, uncovering fibrils of the GalNAc homopolymer that appear to be degraded by endogenous glycohydrolases

**Figure 6:**
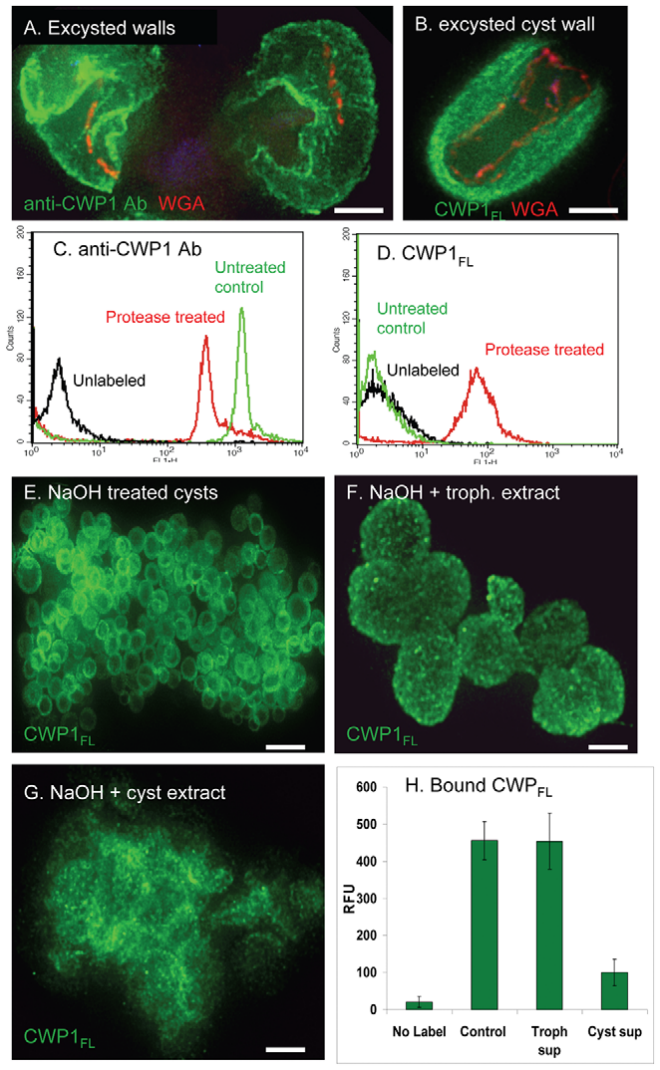
Evidence for the roles of proteases and glycohydrolases in disruption of *Giardia* cyst walls during excystation. A. A representative deconvolving micrograph shows excysted walls of *Giardia*, which were labeled green with anti-CWP1 antibodies, are thickened, have irregular breaks, and appear soft (arrow) and moth-eaten when compared with a sonicated cyst wall (Fig. 1B). B. An excysted wall is extensively labeled green with CWP1_FL_, suggesting the fibrils of the GalNAc homopolymer are exposed by removal of cyst wall proteins by proteases. C and D. Flow cytometry shows that treatment of heat-killed *Giardia* cysts with chymotrypsin decreases the binding of anti-CWP1 antibody (C) but increases the binding of Alexafluor-conjugated CWP1_FL_. E. Representative deconvolving micrograph shows spherical NaOH-treated cyst walls (deproteinated fibrils of the GalNAc homopolymer) labeled green with Alexafluor-conjugated CWP1_FL_. F. These deproteinated cyst walls maintain their spherical shape after incubation with extracts of trophozoites. G. In contrast, NaOH-treated cyst walls, which were incubated with extracts of *Giardia* cysts, lose their spherical shape and form amorphous aggregates. H. These results were confirmed using fluorimetry of NaOH-treated cysts that were incubated with trophozoite and cyst extracts and then labeled with Alexafluor-conjugated CWP1_FL_. All figures are three-dimensional micrographs. Bars (A and B) are 3 microns. Bar (E) is 20 microns. Bars (F and G) are 5 microns.

We chose to use *Giardia* cysts isolated from gerbil infections to study excystation, because the *in vivo* cysts excyst much more efficiently than do cysts made *in vitro* [22], [23]. Excystation *in vitro* has been shown to be dependent upon treatment with trypsin or chymotrypsin, a result that we repeated here (data not shown) [23]. Four changes in the *Giardia* cyst wall occur during excystation:

- Compared to walls of intact cysts stained with anti-CWP1 antibodies, excysted walls are thicker and “fuzzier” in their appearance (compare Fig. 1B to Fig. 6A).
- Excysted walls appear softer and more deformable than intact or sonicated cyst walls (see arrow in Fig. 6A).
- During excystation, fibrils of the GalNAc homopolymer, which can be stained with recombinant CWP1_FL_, become much more accessible (compare Fig. 4B to Fig. 6B).
- Large portions of the walls of excysted organisms disappear, using either the anti-CWP1 antibodies that detects protein (Fig. 6A) or recombinant CWP1_FL_ that detects fibrils of the GalNAc homopolymers (Fig. 6B).

These results suggest that during excystation proteases degrade CWPs and then glycohydrolases degrade fibrils of the GalNAc homopolymer. This two step model for excystation was tested in the following two sections.

### Chymotrypsin removes CWP1 from heat-killed cysts and exposes fibrils of the GalNAc homopolymer

The goal here was to determine whether exogenous chymotrypsin degrades CWPs and exposes fibrils of the GalNAc homopolymer. To test this idea, gerbil-derived cysts were heat-killed by treatment at 56°C for 20 min and then incubated for in 1 mg/ml chymotrypsin at 37°C for 30 min (the same treatment for excystation) prior to washing and labeling with anti-CWP1 antibody (protein label) or recombinant CWP1_FL_ (GalNAc homopolymer label). A negative control was heat-killed cysts that were not treated with chymotrypsin. Flow cytometry showed there is a substantial decrease in labeling with anti-CWP1 antibody after chymotrypsin treatment and a concomitant marked increase in labeling with recombinant CWP1_FL_ (Figs. 6C and 6D). These results echo the effect of NaOH treatment on *Giardia* cyst walls, in which anti-CWP1 antibody binding is eliminated (Fig. 2B) and binding of recombinant CWP1_FL_ is dramatically increased (Fig. 4G).

### Deproteinated fibrils of the GalNAc homopolymer are hydrolyzed by extracts of encysting *Giardia*

To further explore the possibility that *Giardia* contain endogenous glycohydrolases capable of degrading fibrils of the GalNAc homopolymer, we incubated deproteinated fibrils of the GalNAc homopolymer with extracts of trophozoites or with extracts of encysting *Giardia* (Figs. 6E to 6H). Deproteinated fibrils of the GalNAc homopolymer incubated with trophozoite extracts maintain their spherical shape when labeled with recombinant CWP1_FL_ (Figs. 6E and 6F). In contrast, extracts of encysting *Giardia* degrade deproteinated fibrils of the GalNAc homopolymer, so that they form amorphous aggregates that are no longer composed of distinct spheres (Fig. 6G). In addition, the pellet of NaOH-treated cyst walls is much smaller after incubation with the cyst extracts. These results with the deconvolving microscope, which demonstrate a stage-specific glycohydrolase capable of degrading deproteinated fibrils of the GalNAc homopolymer, were confirmed using a fluorimeter (Fig. 6H). In addition, we used GC-MS to demonstrate the release of GalNAc from NaOH-treated walls incubated with cyst extracts (Supplemental Fig. S1C).

## Discussion

### Incorporation of the new findings into a two-component (curled fibril and lectin) model of the *Giardia* cyst wall

**Figure 7:**
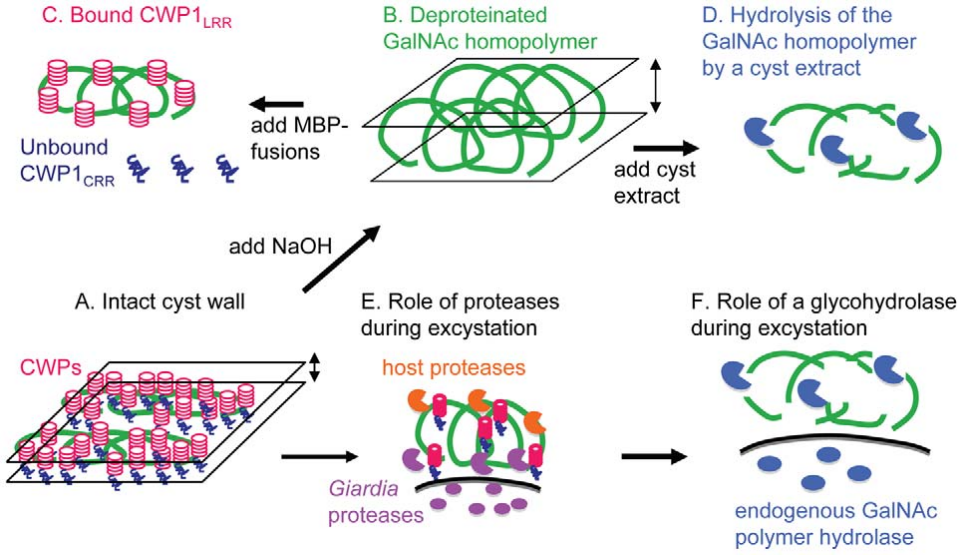
New models for the structure of the *Giardia* cyst wall and for the mechanism of its disruption during excystation based upon the data presented here. A. In the intact *Giardia* cyst wall, curled fibrils of the GalNAc homopolymer are compressed into a narrow plane by bound CWPs (see also Fig. 1). B. When CWPs are removed by hot alkali, curled fibrils of the GalNAc homopolymer form a markedly thickened wall (see also Fig. 2). C. *Giardia* CWPs bind via the *N*-terminal Leu-rich repeats (CWP_LRR_) to fibrils of the GalNAc homopolymer (see also Figs. 3 and4 ). In contrast, the C-terminal Cys-rich regions of CWPs (CWP_CRR_) do not bind to fibrils of the GalNAc homoplymer. D. Deproteinated fibrils of the GalNAc homopolymer are degraded by putative glycohydrolases present in cyst extracts (see also Fig. 6). Disruption of the cyst wall during excystation appears to occur by at least two mechanisms. E. Host and *Giardia* proteases degrade CWPs, which increases the thickness and softens the cyst wall (see also Fig. 6A) [23]. F. Fibrils of the GalNAc homopolymer, which are exposed by proteolysis of CWPs (Figs. 6B to 6D), are degraded by a cyst-specific glycohydrolase (see also Figs. 6E to 6H). As noted in the Introduction and the Discussion, other Cys-rich proteins may be present in the *Giardia* cyst wall [16], [17], and CWPs may be cross-linked by disulfides and be modified by a novel transglutaminase and protein phosphatases [24] to [26].

The intact *Giardia* cyst wall, which is thin, brittle, and impermeable, is composed of fibrils of the GalNAc homopolymer and CWPs (Figs. 2 and 7A). Components of *Giardia* cyst walls studied here (CWPs and fibers of the GalNAc homopolymer) were identified by previous investigators [5]–[9], [24], [25], [27]. What is new is the use of ruthenium red to stain thin sections of intact *Giardia* cyst walls for TEM and use of NANO-W to negatively stain fibrils of the GalNAc homopolymer in sonicated cyst walls (Fig. 1) [43], [44]. Ruthenium red stain demonstrates linear arrays of oval-shaped protein complexes that coat fibrils of the GalNAc homopolymer (Fig. 1C). NANO-W stain demonstrates curled fibrils of the GalNAc homopolymer, which are compressed into a narrow plane by bound CWPs (Figs. 1D and 7A). To our knowledge, there is no other wall (fungal or parasite), which is made on the principle of curled, interlocking fibrils that are pressed into a narrow plane by adherent proteins. While we cannot rule out the possibility that there are cross-links between fibrils of the GalNAc homopolymer, as described from glucans and chitin in fungal walls [33], [42], our images do not show evidence for branching of the deproteinated fibrils.

Treatment with strong base that has previously been used to deproteinate fungal walls effectively removes proteins from cyst walls of *Giardia* (Figs. 2, 3, and 7B) [40]. This is an important improvement over treatment of *Giardia* cyst walls with detergents and proteases, which is time consuming and ineffective [28]. Fibrils of the GalNAc homopolymer prepared by NaOH-treatment of *Giardia* cysts have a uniform diameter of 5 nm and form a loose lattice that has the same hollow, spherical shape as untreated cyst walls. While fibrils of the GalNAc homopolymer of *Giardia* are thinner than the ∼10 nm diameter chitin fibrils present in the cyst wall of *Entamoeba* [35], they are thicker than the 3 to 5 nm diameter microfibrils made by cellulose synthases of corn [47]. These microfibrils, which are composed of 36 parallel chains of β-1,3-linked glucose, coalesce into macrofibrils of cellulose that are 50 to 250 nm in diameter. While the fibrils of the *Giardia* GalNAc homopolymer do not appear to coalesce into macrofibrils, we assume they are also built upon parallel chains of the β-1,3-linked GalNAc that that are closely bound to each other by hydrogen bonds or covalent cross-links. These results are then in agreement with the structural studies of the GalNAc homopolymer [28], which concluded that “the highly insoluble nature of the cyst wall does not stem from the conformational properties of a single GalNAc chain, but must be due to strong interchain interactions.”

How the 10 nm diameter fibrils of the GalNAc homopolymer form such long curled fibrils in the *Giardia* cyst wall is not known, but it may depend upon properties of the GalNAc homopolymer synthase that has not yet been molecularly identified but has been partially purified [31]. Recent genetic analyses of *Candida albicans* chitin synthases show that short microcrystalline rodlets that compose the bulk of the cell wall are made by Chs3p, while long-chitin microfibrils are made by Chs8p [48].

Deproteinated *Giardia* cyst walls are much thicker than untreated walls and are no longer brittle and impermeable to small molecules. These results show the thinness, brittleness, and impermeability of the *Giardia* cyst wall depend upon the presence of CWPs. These qualities of the cyst wall likely also depend upon formation of disulfide bonds, isopeptide bonds, and dephosphorylation of CWPs, as suggested by previous studies of encystation by *Giardia* [24 to 27].

The demonstration here that native CWP1 and MBP-fusion proteins containing the full length CWP1 (CWP1_FL_) or the Leu-rich repeat domain of CWP1 (CWP1_LRR_) bind to deproteinated fibrils of the GalNAc homopolymer strongly suggests CWP1 is a novel lectin that binds to fibrils of the unique GalNAc homopolymer (Figs. 3A to 3C, 4B to 4D, 6B, 6D to 6H, and 7C). There is mass spectrometry evidence that native CWP2 also binds to the deproteinated fibrils of the GalNAc homopolymer (Fig. 3D). Mass spectrometry results failed to make a compelling case that CWP3, which is added late to the cyst wall [20], binds to deproteinated fibrils of the GalNAc homopolymer. In addition, we did not have evidence for binding of Cys-rich *Giardia* cyst wall proteins (HCNCp and EGFCPs) to deproteinated fibrils of the GalNAc homopolymer [16], [17].

The Cys-rich domain of CWP1 does not appear to contain the lectin activity that binds to the deproteinated fibrils of the GalNAc homopolymer (Fig. 4F). It is not known whether the Cys-rich domains of other *Giardia* cyst wall proteins (HCNCp and EGFCPs) have lectin activity for the GalNAc homopolymer [16], [17]. In contrast, Cys-rich domains have lectin activities in *Entamoeba* cyst wall proteins and in plant lectins such as WGA [35], [36], [38], [39], [49]. In addition, Leu-rich repeats present in Toll-like receptors and in cytosolic NOD-like receptors recognize peptides, lipids, or nucleic acids rather than carbohydrates [50], [51].

Using recombinant CWP1_FL_ as a novel probe for fibrils of the GalNAc homopolymer, we showed that synthesis of the GalNAc homopolymer occurs within numerous small vesicles in encysting *Giardia* (Fig. 5). This result, which is consistent with TEM observations of fibrils within vesicles of encysting *Giardia*, suggests that the GalNAc homopolymer synthase is not restricted to the plasma membrane, as has been shown for fungal chitin synthases [30], [42]. Using similar methods, we found that *Entamoeba* also synthesizes chitin in vesicles [39], while single-cell algae also synthesize cellulose within intracellular vesicles [52]. Double-labeling experiments here suggest that the fibrils of the GalNAc homopolymer are not made in ESVs that contain CWP2 and CWP3 and are present early on the surface of encysting *Giardia* when epitope-tagged CWP2 and CWP3 are for the most part still present in ESVs (Figs. 5D to 5G). These experiments complement the recent demonstration that CWP1 and the major portion of CWP2 are added first to the cyst wall followed by CWP3 [20].

### A two-component (protease and glycohydrolase) model for excystation

Our experiments suggest that breaking the cyst wall of *Giardia* during excystation likely occurs in at least two conceptual steps. First, exogenous serine proteases (e.g. trypsin and chymotrypsin) degrade CWPs that thickens and softens the cyst wall (Figs. 6A, 6C, and 7E) [23]. As shown previously by inhibition studies, endogenous cysteine proteases of *Giardia* are also involved in excystation [22]. Second, fibrils of the GalNAc homopolymer, which are exposed by proteolysis of CWPs (Figs. 6B, 6D, and 7F), are degraded by an endogenous glycohydrolase. Evidence for the endogenous glycohydrolase of *Giardia* includes disappearance of large portions of the cyst wall (detected by anti-CWP1 antibodies and by CWP1_FL_) during excystation (Figs. 6A and 6B) and demonstration that extracts of *Giardia* cysts are capable of degrading deproteinated fibrils of the GalNAc homopolymer (Figs. 6E to 6H and 7D). The role of stomach acids and protist motility during excystation are not addressed in this model [23].

### Summary and unanswered questions

The major discoveries here are the following: 1) In intact cyst walls of *Giardia*, fibrils of the unique GalNAc homopolymer are curled and form a lattice that is compressed into a narrow plane by bound protein. 2) Leu-rich repeats of CWP1 form a novel lectin domain that is specific for fibrils of the GalNAc homopolymer, which can be isolated by methods used to deproteinate fungal walls [40]. 3) A cyst-specific glycohydrolase is able to degrade deproteinated fibrils of the GalNAc homopolymer.

We understand that our models for *Giardia* cyst wall structure and mechanism of disruption during excystation are oversimplified and incompletely demonstrated. Follow up studies will include identification of proteins present in oval-shaped aggregates that form linear arrays on fibrils of the GalNAc homopolymer. We plan to determine whether and how fibrils of the GalNAc homopolymer interact with CWPs in intracellular vesicles prior to release onto the surface of encysting *Giardia*. In addition, the *Giardia* enzymes that synthesize and degrade the unique GalNAc homopolymer need to be molecularly characterized, and their function tested by knock-in or knock-down methods.

## Materials and Methods

### Ethics statement

Culture and manipulation of *Giardia*, including production of cysts *in vitro* and handling of cysts from gerbils, has been has been approved by the Boston University Institutional Biosafety Committee (BU IBC). Similarly, recombinant expression of *Giardia* proteins in bacteria has been approved by the BU IBC. Monoclonal anti-CWP1 antibodies and cysts of the H3 strain of *Giardia*, which had been passaged through gerbils, are catalog items from a commercial vendor (Waterborne Incorporated, New Orleans, LA).

### Parasites examined

Trophozoites of the WB strain of *Giardia lamblia* (the first genome project strain) were grown axenically in TYI-S-33 medium supplemented with 0.1% bovine bile for 48 hrs at 37°C [2]. Trophozoites were encysted by standard methods [5]. Encystation was induced by exchanging trophozoite medium with TYI-S-33 medium supplemented with 10 mg/ml bile salts, pH 7.8. Non-adherent water-resistant cysts were isolated and washed twice with deionized water. Contaminating fecal bacteria were removed from cysts in fecal material by sucrose and percoll density gradient centrifugation.

### Isolation and biochemical characterization of the GalNAc homopolymer

*Giardia* cysts obtained *in vitro* (∼40 million) were treated with 1 N NaOH for 1 hr at 100°C, insoluble cyst wall material was recovered by centrifugation at 2000 rpm at room temperature for 10 min. This material was then suspended in 0.75 N NaOH and heated to 75°C for 2 hrs. The suspension was then allowed to cool to room temperature and extraction was continued for 16 hrs. The insoluble material was recovered by centrifugation at 2000 rpm at room temperature for 10 min and then washed ten times with deionized water [40].

Proteins associated with the NaOH-treated cyst walls were determined using a micro BCA protein assay kit (Pierce Biotechnology). Alternatively, NaOH-treated cysts were boiled in 1% SDS, and extracted proteins were separated on SDS-PAGE containing 4–20% polyacrylamide. Gels were fixed and stained with silver, or unfixed proteins were transferred to PVDF membranes and probed with anti-CWP1 monoclonal antibody [7]. The latter was detected with anti-mouse secondary antibody conjugated to horse-radish peroxidase and developed with a chemiluminescent substrate. Positive controls for the BCA assay, silver stains, and anti-CWP1 blot were NaOH-treated cysts, which were labeled with recombinant MBP-CWP1 fusion-proteins (see below).

Monosaccharides in the NaOH-treated cyst walls were released with 4 N H_2_SO_4_ at 100°C for 4 hrs; H_2_SO_4_ was neutralized with saturated Ba(OH)_2_ solution; and sugars were concentrated using a rotavap. Released monosaccharides were analyzed on a high performance anion exchange chromatography (HPAEC) with a pulsed amperometric detector in a Dionex LC20 instrument using ED40 gold electrode and an analytical Carbopac MAI column (250×4 mm) equilibrated in 100 mM NaOH. The flow rate was 0.4 ml/min with 100 mM to 800 mM sodium hydroxide gradient. GalNAc, GlcNAc, GalN, GlcN, and Glc were used as standards. Alternatively, released monosaccharides were analyzed by GC/MS (next section).

### GC-MS analysis of carbohydrates in deproteinated cyst walls

NaOH-extracted *Giardia* cyst walls were dissolved in 500 µl of 1 M methanolic-HCl at 90°C for 16 h. The re-N-acetylation of the monosaccharides mixture was performed by adding 500 µl of methanol, 10 µl of pyridine and 50 µl of acetic anhydride at room temperature for 15 min. Sugars were trimethylsilylated in 200 µl of N,O-bis-(trimethylsilyl)-acetamide (TMSA) at 90°C for 15 min. The sample was dried down under nitrogen, dissolved in 50 µl of hexane and centrifuged to remove the excess of solid reagents. The hexane supernatant (1/60) was used for the GC-MS analysis.

GC-MS analyses were performed on a Hewlett-Packard 5890 instrument, in the following conditions: ZB-5 capillary column (Phenomenex, 30 m×0.25 mm i.d., flow rate 0.8 ml/min, He as carrier gas). The injection temperature was 250°C. For sugar analyses the oven temperature was increased from 25°C to 90°C in 1 min and held at 90°C for 1 min before increasing to 140°C at a rate of 25°C/min, to 200°C at 5°C/min and finally to 300°C at 10°C/min. Electron ionisation (EI) mass spectra were recorded by continuous quadrupole scanning at 70 eV ionisation energy.

### Transmission electron microscopy (TEM) of *Giardia* cyst walls

*Giardia* cysts were treated with NaOH, and deproteinated cyst walls were isolated by centrifugation and washed in PBS. Alternatively, *Giardia* cysts were treated with 200 pulses from a probe sonicator in PBS on ice, and cyst walls were purified by centrifugation through a 60% sucrose cushion, using methods similar to those used to prepare *Entamoeba* cyst walls [35], [38]. For negative-staining, NaOH-treated cyst walls or cyst walls isolated on sucrose gradients were washed and resuspended in sterile deionized water and applied to carbon-coated, copper grids. The grids were stained with 1% uranyl acetate for 15 sec and visualized on a Phillips CM-12 microscope at 80 KV, 3800 to 17,000 times magnification. Alternatively, grids were stained with 2% Nano-W solution in water at pH 6.8 for 30 sec [44]. Images were recorded on Kodak 50–63 film and digitized on a Nikon 9000 scanner at 2000 dpi.

For conventional transmission microscopy, cyst walls were fixed in 2% paraformaldehyde and 1% gluteraldehyde buffered by 0.1 M sodium cacodylate, pH 7.3, for 12+ hrs at 4°C for conventional TEM. Cyst walls were post-fixed for 12+ hrs at 4°C in 1% osmium tetroxide +/− 0.5 mg/ml ruthenium red in cacodylate buffer [43]. Water was removed from pellets in graded ethanols and propylene oxide, and organisms were embedded in Epon at the Harvard Medical School Electron Microscopy Facility. Ultrathin sections (∼60–80 nm) were cut on a Reichert Ultracut-S microtome, picked up onto copper grids, stained with 1% ruthenium red and osmium tetroxide and examined in a “Tecnai G^2^ Spirit BioTWIN” transmission electron microscope. Images were taken with a 2k AMT CCD camera.

### Recombinant expression of *Giardia* cyst wall proteins in transformed bacteria

The full length *Giardia* CWP1 gene (CWP1_FL_) minus the 5′ end that encodes the N-terminal signal sequence was PCR amplified using a sense primer (GGAATTCCTCACTTGCCCGGCTACTC) and an anti-sense primer (GCTCTAGAAGGCGGGGTGAGGCAGTA). *The* N*-terminal Leu-rich repeat of CWP1 (CWP1_LRR_) was amplified using the same sense primer and a new anti-sense primer (GCTCTAGAGTTGAGATAGAGCTCCATAAGGTAGG). The C-terminal Cy-rich region of CWP1 (CWP1_CRR_) was amplified using a new sense primer (GGAATTCCTCTATCTCAACTGCAACCCTGA) and anti-sense primer for CWP1_FL_. The three* Giardia *CWP1 PCR products were cloned into pMAL-p2E vector (New England Biolabs (NEB), Beverley, MA), which makes an IPTG-inducible, periplasm-targeted fusion-protein with MBP at the* N*-terminus and CWP1 at the C-terminus [41]. Bl21-DE3 cells from Invitrogen were transformed with the pMAL-p2E- constructs, and recombinant proteins were induced with 0.1 mM IPTG.* E. coli *expressing MBP fusion proteins in the periplasm were suspended in 20% sucrose in PBS for 10 min in ice. After removing sucrose solution, periplasmic proteins were released by hypotonic shock in phosphate-buffer (PB) and 4 mM PMSF on ice. Fusion-proteins were purified by amylose resin (NEB) using 50 mM maltose as an eluent. Enriched MBP-CWP1 fusion-proteins were checked for purity on SDS-PAGE stained with Coomassie brilliant blue and by Western blot using horse-radish peroxidase-tagged anti-MBP antibody from NEB.*

### Deconvolving microscopy

For labeling the surface of *Giardia* red, trophozoites and encysting organisms were incubated for 60 min at 4°C in 200 mM carbonate-bicarbonate buffer, pH 9.3, with 20 mg/ml of the amine-reactive probe Alexa Fluor 610-X, succinimidyl ester, bis(triethylammonium salt) 6-isomer (Molecular Probes, Invitrogen) [46]. Alexafluor-labeled trophozoites were washed three times with PBS to remove excess fluorochrome, and parasites were fixed and in 2% paraformaldehyde for 5 min at 4°C and permeabilized with 0.1% Triton X-100 in PB for 1 min at 4°C temp. Alternatively, *N*-glycans on glycoproteins of fixed and permeabilized *Giardia* were labeled red using WGA conjugated with TRITC (EY laboratories), using methods described [16]. NaOH-treated cyst walls, which were used without fixation, were also labeled with WGA-TRITC to detect residual glycoproteins.

Recombinant MBP-CWP1 fusion-proteins were labeled green with Alexafluor 488 carboxylic acid, succinimidyl ester (Molecular Probes, Invitrogen) in 200 mM carbonate-bicarbonate buffer, pH 9.3, and unreacted fluorochromes were removed by dialysis versus PBS. Fixed and permeabilized *Giardia* or NaOH-treated cyst walls were incubated with 0.05 to 0.1 mg/ml of each fusion-protein for 1hr at room temperature in PBS and washed 3 times in PBS. Prior to deconvolving microscopy, nuclei were stained blue with 0.1 mg/ml DAPI.

Mouse anti-CWP1 monoclonal antibodies, which were labeled with fluorescein, were purchased from Waterborne Incorporated [7], [11]. Antibodies were diluted 1:200 and incubated with fixed and permeabilized *Giardia* or NaOH-treated walls for 1 hr at RT in PBS. One percent bovine serum albumin (BSA) in PBS was used as a blocking reagent. Preparations were washed three times in PBS.

Double-labeling experiments were performed with transformed *Giardia* using vectors designed to express epitope-tagged CWP2 or CWP3 during encystation that were a generous gift of Adrian Hehl [20]. Briefly, these vectors were linearized, and *Giardia* were transformed by electroporation and selected with neomycin or puromycin. CWP2 had a FLAG-tag at the N-terminus that was detected with an anti-FLAG antibody conjugated to Alexafluor-594. CWP3 had a GFP-tag at the C-terminus that was detected with an anti-GFP antibody conjugated to Alexafluor-594. Recombinant CWPFL conjugated to Alexafluor-488 was used to detect the GalNAc homopolymer. Double-labeling experiments were performed on *Giardia* trophozoites and organisms encysting for 6 to 12 hrs.

Slides were examined by three-dimensional multiple wavelength fluorescence microscopy using an Olympus IX70 microscope equipped for Deltavision deconvolution (Applied Precision). This system employs restorative as well as deconvolution techniques to provide resolutions up to four times greater than conventional light microscopes [53]. Images were collected at 0.2 mm optical sections for the indicated wavelengths and were subsequently deconvolved using SoftWoRx (Applied Precision). Data were examined as either optical sections or as a projection of the entire stack.

### Flow cytometry

Intact gerbil-derived cysts were labeled with Alexafluor-488-conjugated to anti-CWP1 antibody or to MBP-CWP1_FL_ and then examined with an FCCF FACSCaliber flow cytometer using BD Cellquest Pro v5.2 software (Becton Dickinson). NaOH-treated cyst walls (also referred to a deproteinated fibrils of the GalNAc homopolymer were also labeled with anti-CWP1 antibody or MBP-CWP1_FL_ and then examined with the flow cytometer. A third labeling experiment used gerbil-derived cysts that were heat-killed by treatment at 56°C for 20 min and then incubated for in 1 mg/ml chymotrypsin at 37°C for 30 min prior to washing and labeling with anti-CWP antibody or recombinant CWP1_FL_.

### Identification of a GalNAc homopolymer hydrolase activity in extracts of *Giardia* trophozoites and cysts

Protein extracts from *Giardia* trophozoites and from *Giardia*, which had encysted for 24 to 48 hrs, were prepared by lysing *Giardia* in PBS with 0.1% Triton X -100 and 10 µm E64 to inhibit Cys-proteases. Fibrils of the GalNAc homopolymer, which had been deproteinated with NaOH, were incubated extracts from trophozoites or cysts for 18 hrs at 37°C. Hydrolysis of the GalNAc homopolymer by the protein extracts was determined by three methods. First, residual fibrils of the GalNAc homopolymer were labeled with Alexafluor-conjugated CWP1_FL_ and examined with the deconvolving microscope. Second, the relative amount of Alexafluor-conjugated CWP1_FL_ bound to fibrils of the GalNAc homopolymer treated with each protein extract was compared to that bound to untreated fibrils of the GalNAc homopolymer using a Tecan Spectrafluor plus fluorimeter. Third, release of GalNAc from fibrils of the GalNAc homopolymer treated with the cyst extract into the supernatant was determined by GC-MS essentially as described above for analysis of monosaccharides released from deproteinated fibrils of the GalNAc homopolymer by treatment with strong acid.

## Supporting Information

Figure S1 NaOH-treated cyst walls of *Giardia* are composed of GalNAc, and GalNAc is released from these NaOH-treated cyst walls by glycohydrolases present in extracts of encysting parasites. A. GC-MS of monosaccharides produced by acid treatment of NaOH-treated cyst walls, followed by reacetylation, show only GalNAc. B. HPAEC of similar material shows GalN (acid deacetylates GalNAc). C. GC-MS shows that GalNAc is released from NaOH-treated cyst walls treated with an extract of encysting *Giardia*.
(1.45 MB TIF)
