# Supplementary figures and images for: Giardia Cyst Wall Protein 1 Is a Lectin That Binds to Curled Fibrils of the GalNAc Homopolymer
Source: PLoS Pathog. 2010 Aug 19;6(8):e1001059. doi: 10.1371/journal.ppat.1001059 (PMC2924369; doi:10.1371/journal.ppat.1001059)

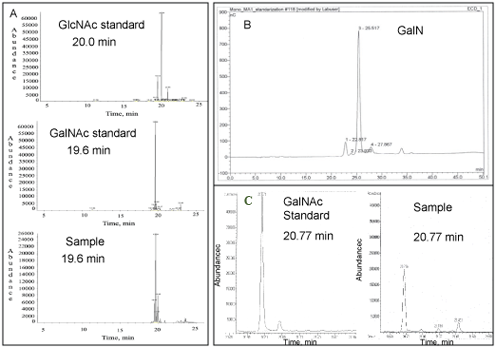

Supplement: Figure S1 — NaOH-treated cyst walls of Giardia are composed of GalNAc, and GalNAc is released from these NaOH-treated cyst walls by glycohydrolases present in extracts of encysting parasites. A. GC-MS of monosaccharides produced by acid treatment of NaOH-treated cyst walls, followed by reacetylation, show only GalNAc. B. HPAEC of similar material shows GalN (acid deacetylates GalNAc). C. GC-MS shows that GalNAc is released from NaOH-treated cyst walls treated with an extract of encysting Giardia. (1.45 MB TIF) [file ppat.1001059.s001.tif]
